# Methodological considerations in interpreting virtual reality-assisted analgesia during nasal procedures – a letter to the editor

**DOI:** 10.1097/MS9.0000000000005069

**Published:** 2026-04-29

**Authors:** Ibad Ur Rahman, Kamil Ahmad Kamil, Hashir Zia Haider, Samiullah Khan

**Affiliations:** a Department of Medicine, Khyber Medical College, Peshawar, Pakistan; b Internal Medicine Department Mirwais Regional Hospital, Kandahar, Afghanistan

**Keywords:** nasal endoscopy, otolaryngology, procedural pain, virtual reality

## Abstract

A recent randomized-controlled trial by Alotaibi *et al* investigated the use of virtual reality (VR) for pain and anxiety management during nasal procedures, reporting positive findings that warrant critical methodological examination. While the study’s randomized design strengthens internal validity, the interpretation of its findings is complicated by the inclusion of an intra-group analysis comparing current VR-assisted pain to patients’ recalled pain from unstandardized historical procedures, which introduces a high risk of recall bias. Furthermore, the unblinded nature of the study makes the self-reported outcomes susceptible to expectancy and novelty effects, potentially overestimating the true treatment benefit. Although VR holds significant potential as a non-pharmacological analgesic in otorhinolaryngology, the findings must be interpreted with caution, and future investigations should prioritize standardized controls and objective measures to accurately determine its independent clinical effect on procedural pain.

*To the Editor,*

This letter to the editor adheres to the TITAN guideline on the need for transparency in AI use in healthcare^[^1^]^.We read with interest the randomized-controlled trial by Alotaibi et al. examining the effects of virtual reality (VR) on pain, anxiety, and patient satisfaction during office-based otorhinolaryngology procedures^[^2^]^. The exploration of non-pharmacological strategies to improve procedural tolerance is increasingly important as immersive technologies receive greater attention in pain management^[^3–5^]^.

Randomized allocation to the VR and control groups enhances internal validity; however, several limitations need clarification when interpreting the findings. Besides randomized comparisons, the study presents intra-group analyses within the VR arm, comparing pain experienced during the current VR-assisted procedure to pain recalled from previous procedures without VR. These historical procedures were not standardized regarding timing, operator, or clinical environment. Consequently, retrospective comparisons are vulnerable to recall and contextual bias. In addition to this, previous research also suggests that recollections of procedural pain are influenced by cognitive and emotional factors, which may result in overestimation of the perceived treatment benefit^[^6^]^.

This issue is particularly pertinent because all primary outcomes – pain intensity, anxiety, and patient satisfaction – were assessed using self-reported measures in an unblinded context. Due to the immersive nature of VR interventions, participant blinding was not feasible. Participant awareness of receiving a novel technology may introduce expectancy or novelty effects, which can influence the reporting of outcomes. Evidence from randomized trials of VR-based distraction demonstrates that psychological engagement and attentional shifts contribute to perceived pain relief, making perception-based results particularly susceptible to expectation effects^[^4,5,7^]^.

These methodological considerations acknowledge the potential of VR to enhance patient comfort during nasal procedures, while emphasizing the necessity for cautious interpretation of the current findings. They emphasize the importance of future research using standardized comparisons, blinded assessments when possible, and objective outcome measures. Conducting larger multicenter trials utilizing these methodologies may more accurately determine the independent effects of immersive VR on procedural pain and patient experience in routine otorhinolaryngology practice^[^8^]^.

## Data Availability

All data used are from publicly available published sources.
